# Machine learning to predict metabolic drug interactions related to cytochrome P450 isozymes

**DOI:** 10.1186/s13321-022-00602-x

**Published:** 2022-04-15

**Authors:** Ning-Ning Wang, Xiang-Gui Wang, Guo-Li Xiong, Zi-Yi Yang, Ai-Ping Lu, Xiang Chen, Shao Liu, Ting-Jun Hou, Dong-Sheng Cao

**Affiliations:** 1 Department of Pharmacy, Xiangya Hospital, Central South University, Changsha, 410008 Hunan People’s Republic of China; 2 National Clinical Research Center for Geriatric Disorders, Xiangya Hospital, Central South University, Changsha, 410008 Hunan People’s Republic of China; 3 Xiangya School of Pharmaceutical Sciences, Central South University, Changsha, 410013 Hunan People’s Republic of China; 4 Eye Center of Xiangya Hospital, Central South University, Changsha, 410008 Hunan People’s Republic of China; 5 Advancing Translational Medicine in Bone and Joint Diseases, School of Chinese Medicine, Hong Kong Baptist University, Hong Kong, SAR People’s Republic of China; 6 Department of Dermatology, Hunan Engineering Research Center of Skin Health and Disease, Hunan Key Laboratory of Skin Cancer and Psoriasis, Xiangya Hospital, Central South University, Hunan Changsha, People’s Republic of China; 7 Innovation Institute for Artificial Intelligence in Medicine of Zhejiang University, College of Pharmaceutical Sciences, Zhejiang University, Hangzhou, 310058 Zhejiang People’s Republic of China

**Keywords:** Metabolic drug interaction, CYP450, Machine learning, Drug combination, Adverse drug reactions

## Abstract

Drug–drug interaction (DDI) often causes serious adverse reactions and thus results in inestimable economic and social loss. Currently, comprehensive DDI evaluation has become a major challenge in pharmaceutical research due to the time-consuming and costly process of the experimental assessment and it is of high necessity to develop effective in silico methods to predict and evaluate DDIs accurately and efficiently. In this study, based on a large number of substrates and inhibitors related to five important CYP450 isozymes (CYP1A2, CYP2C9, CYP2C19, CYP2D6 and CYP3A4), a series of high-performance predictive models for metabolic DDIs were constructed by two machine learning methods (random forest and XGBoost) and 4 different types of descriptors (MOE_2D, CATS, ECFP4 and MACCS). To reduce the uncertainty of individual models, the consensus method was applied to yield more reliable predictions. A series of evaluations illustrated that the consensus models were more reliable and robust for the DDI predictions of new drug combination. For the internal validation, the whole prediction accuracy and AUC value of the DDI models were around 0.8 and 0.9, respectively. When it was applied to the external datasets, the model accuracy was 0.793 and 0.795 for multi-level validation and external validation, respectively. Furthermore, we also compared our model with some recently published tools and then applied the final model to predict FDA-approved drugs and proposed 54,013 possible drug pairs with potential DDIs. In summary, we developed a powerful DDI predictive model from the perspective of the CYP450 enzyme family and it will help a lot in the future drug development and clinical pharmacy research.

**Graphical Abstract:**

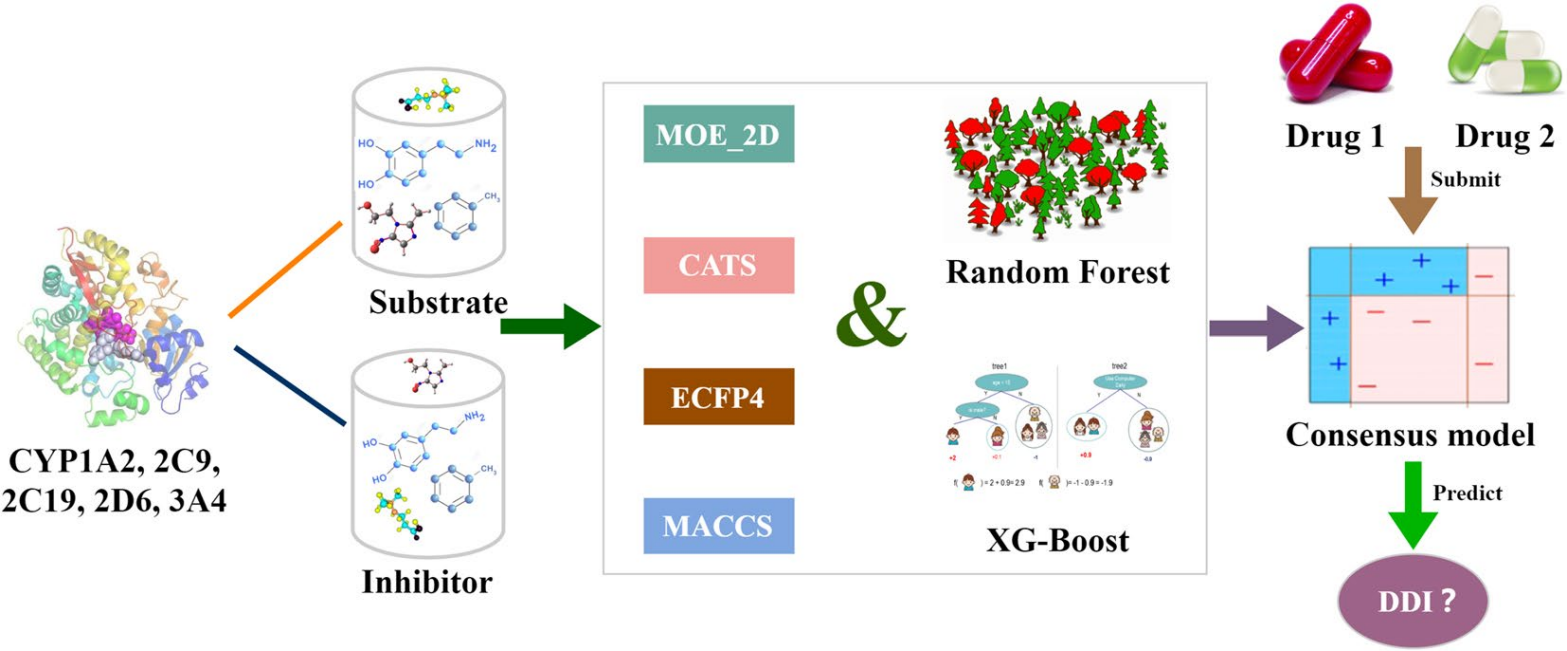

**Supplementary Information:**

The online version contains supplementary material available at 10.1186/s13321-022-00602-x.

## Introduction

With the increasing complexity of clinical diseases and the rapid development of pharmaceutical industry in recent years, multi-drug combination has become a common and promising treatment option for doctors and pharmacists. However, in addition to effectively treating diseases, drug combinations also greatly increase the risk induced by drug interactions. Clinically, if a drug is co-administered with another or more drugs, drug–drug interactions (DDIs) may occur, which may affect the efficacy or/and safety of this drug [1]. Therefore, in-depth understanding of DDIs is of high importance for enhancing synergistic effects of drugs and reducing adverse drug reactions [2]. A survey between 2010 and 2011 showed that 67% of older Americans were using five or more different medications at the same time, including prescription, over-the-counter and health supplements, etc*.* [3]. The ideal result of drug combination is to improve the treatment effect, but in fact, numerous adverse reactions or even serious side effects were caused by drug combination, which makes DDIs be a major problem in medical process. In China, more than 100,000 people die due to severe adverse reactions every year, making it the fourth leading cause of deaths. Among these, unpredictable DDIs contribute to about 30% of the reported adverse drug reactions [4, 5]. In addition, serious adverse reaction caused by DDI is also one of the main reasons for the drug withdrawal. Therefore, how to detect the possible adverse effects caused by DDIs as early as possible before the clinical use is an important topic in clinical practice.

DDIs can be divided into two categories according to their mechanisms: pharmacodynamics-based DDI and pharmacokinetics-based DDI. The former usually occurs when two or more drugs acting on the same or similar receptor at the same time, which may result in stronger pharmacodynamic effects (additive or synergistic) or reduce the efficacy of drugs (antagonistic). The latter usually occurs when one drug changes the absorption, distribution, metabolism and excretion (ADME) of the co-administered drugs [6]. Currently, pharmacokinetic-based DDIs have the highest clinical incidence, and involves various enzymes and transporters, among which cytochrome P450 (CYP450) is the most important phase I metabolism enzyme family for human, and has 57 functional genes. It is reported that more than 2/3 of xenobiotics are metabolized by the CYP450 enzyme family, and 80% of them are metabolized by five isozymes, namely CYP1A2, CYP2C9, CYP2C19, CYP2D6 and CYP3A4 [7]. Therefore, the CYP450 enzyme family, especially aforementioned five isozymes play a vital role in the drug metabolism. Clinically, the metabolic DDIs based on CYP450 isoenzymes are very common, and 70% of them are caused by enzyme inhibition. That is to say, one drug can change the metabolic characteristics of another drug by inhibiting a specific subtype of the CYP450 enzyme family, leading to adverse drug interactions. This type of DDIs extensively occurs in the clinical application of drugs, such as theophylline and ciprofloxacin (substrate and inhibitor of CYP1A2), warfarin and ibuprofen (substrate and inhibitor of CYP2C9), phenytoin sodium and fluvoxamine (substrate and inhibitor of CYP2C19), terfenadine and ketoconazole (substrate and inhibitor of CYP3A4), and so on. Considering these clinical phenomena, we hold the opinion that it is crucial to evaluate drug metabolic interactions based on CYP450 enzyme family before the initiation of clinical drug combinations.

Currently, there are two traditional approaches to study drug metabolic interactions based on CYP450: experimental methods and computational methods. Experimental methods mainly include some in vitro approaches such as the primary hepatocyte culture method, the liver microsomal method, the liver biopsy method and the gene recombination method; the in vivo methods mainly refer to animal experiments and the probe drug method. However, all the aforementioned methods are time-consuming and need extensive investment. With the development of artificial intelligence (AI) and accumulation of experimental data, computational approach has become an important way to study DDIs. Currently, computational DDI studies can be divided into two categories: network-based and quantitative structure–activity relationships (QSAR)-based. Network-based approaches aim to analyze the targets and pathways affected by side effect-related drugs. With the development of network medicine, scientific approaches to analyze and predict DDIs at the molecular level have emerged. For example, in 2011, Murat Iskar et al*.* have realized the prediction of drug interactions by integrating molecular and pharmacological data of drug pairs. Further analysis confirmed that among the top ranked predictions, 69% could be supported by literatures [8]. Takarabe et al*.* have also developed a drug interaction retrieval system in the KEGG DRUG database, which may be used for both searching against known drug interactions and predicting potential interactions [9]. In 2013, Huang et al*.* constructed a protein–protein interaction network based on 1249 FDA-approved drugs including 1289 targets and 4776 relations [10]. In this study, researchers collected and integrated the pharmacokinetic and clinical characteristics of the drugs, so that the predictive ability of the model was better than those of the models using a single type of data. Additionally, Guimera et al*.* also used the large-scale unsupervised network reasoning method to speculate potential drug interactions [11]. In 2017, Takeda et al. studied the effect of two-dimensional similarity on drug interactions based on the network of pharmacokinetics and pharmacodynamics, and they finally built a logistic regression model to effectively predict drug interactions [12, 13]. QSAR-based approaches aim to establish QSAR models for DDIs. For example, Vilar et al. established a model based on structural similarity using the MACCS and interaction profile fingerprints (IPF) to predict drug interactions with the sensitivity of 0.68 and the specificity of 0.96 [14, 15]. Most recently, Dmitriev et al. applied the Prediction of Activity Spectra for Substances (PASS) software and Pairs of Substances Multilevel Neighborhoods of Atoms (PoSMNA) descriptors to build a series of QSAR models for DDIs mediated by the seven most important P450 cytochromes and obtained satisfactory results [16]. However, there are several issues that could be improved, such as no profound mechanism discussion, smaller modeling dataset and inadequate external validation. In addition to the above studies, Percha and Tari also employed the text mining method in 2010 and 2012 respectively to establish the prediction models of drug interactions, and the prediction accuracy reached about 80% [17, 18]. In 2015, Zhang et al. built a drug interaction predictive model based on clinical side effects integrated from drug labeling and FDA adverse reaction reporting system. They predicted drug interactions among 1626 compounds and predicted 145,068 drug interactions to help clinicians avoid high-risk drug combinations when prescribing [19].

To sum up, although a lot of work have been done for predicting drug interactions and some have focused on metabolic DDI [20–24], they still have obvious shortcomings: (1) many suppositions about DDI prediction were based on multiple different assumptions, and thus multiple uncertainties greatly reduce the accuracy and reliability of the predictive models; (2) Most of the reported studies did not involve a specific DDI mechanism and thus the intrinsic rules were still unknown; (3) DDI researches related CYP450 lacked profound mechanism discussion and adequate modeling dataset and external validation. Based on this phenomenon, we took metabolic DDI, which is very important in drug–drug interactions, as the pointcut for our study. In this paper, a simple and specific mechanism was proposed for subsequent in-depth DDI prediction research and mechanism discussion. Not only that, a series of relatively larger datasets, two state-of-art machine learning methods and a systematic external validation procedure were prepared for model building and validation. Furthermore, related DDI mechanism discussion based on scaffold analysis and comparison with recent published models were carried out to reveal the intrinsic rules of DDI and test the robustness of our models. Detailed steps were described as follows: Firstly, we manually collected the substrates and inhibitors for five important CYP450 isozymes and checked them carefully for further modeling. Secondly, we constructed the predictive models based on different types of descriptors and machine learning algorithms, and then developed a consensus model with satisfactory predictive ability. Thirdly, we further evaluated the predictive ability of the consensus DDI model using the external datasets and multi-level validation. Fourthly, the scaffold analysis and comparison with recent published models were carried out to prove the reliability and effectiveness of our models. Finally, we applied our DDI model to FDA-approved drugs in order to provide some clues to help clinicians avoid high-risk drug combinations in prescribing.

## Research hypothesis

Generally, when patients take two drugs at the same time and both drugs interact with the same CYP450 isozyme (substrate or inhibitor), metabolic interactions may occur. As shown in Table 1, we can draw the conclusion that if two drugs are substrates and inhibitors of the same CYP450 isozyme respectively, they may cause changes in plasma concentrations to different degrees when used together. Based on this phenomenon, we proposed the research hypothesis for this study: If two drugs are substrates and inhibitors of the same CYP450 isozyme, metabolic drug interactions will occur when the two drugs are combined. Based on this hypothesis, we will construct several DDI prediction models for five important CYP450 isozymes and aim to develop a more accurate and rapid assessment approach for metabolic DDIs that can better serve drug discovery and help clinicians avoid high-risk drug combinations in prescribing.

**Table 1:** Changes of the plasma concentration when two drugs are combined

Drug 1/2	Inhibitor	Substrate	Both
Inhibitor	Affect other drugs metabolized by the isozyme	C_p_ of Drug 1 will increase	C_p_ of Drug 1 will increase
Substrate	C_p_ of Drug 2 will increase	If bind the same active site, C_p_ of Drug 1, 2 will increase	C_p_ of Drug 1, 2 will increase
Both	C_p_ of Drug 2 will increase	C_p_ of Drug 1, 2 will increase	C_p_ of Drug 1, 2 will increase

C_p_: the drug concentration in plasma

## Materials and methods

### Data collection

#### Modeling data

*Positive dataset* we collected the positive dataset (the substrates or inhibitors of five CYP450 isoenzymes) from three sources: Firstly, we searched for the human UniProt ID of the five CYP450 isoenzymes in the UniProt database and then found the “Drug relation” module in the DrugBank database according to their UniProt ID. After that, we manually collected the substrates and inhibitors of five isozymes. After checking the structures, we reserved the drug compounds with specific information. Secondly, we further manually collected the substrate and inhibitor data of five isozymes in the “CYP-Drug interaction” module of the SuperCYP database, and reserved human data with chemical structures. Thirdly, we searched the bioassay record “AID 1851” in the PubChem database and downloaded the original inhibitor dataset, which contained heterogeneous information about the inhibition test for five important CYP450 enzymes (CYP1A2, CYP2C9, CYP2C19, CYP2D6, CYP3A4). We preserved several types of important information for our following study and then five new datasets including PUBCHEM_CID and Activity Outcome (label “active”) for each enzyme were created. Corresponding chemical structures (SMILES) were obtained from PubChem database based on their PUBCHEM_CID. The SMILES structures of these molecules were checked one by one to ensure their correctness and solvent or saline ions adhering to the molecules were removed automatically by MOE software. So far, five well-organized inhibitor datasets including drug structures were prepared. Similarly, for these inhibitor data collected from DrugBank and SuperCYP, only structure information was carried. If there were conflicting labels for one molecule while combining data from different sources, we will comfirm it again. Molecules that can be ensured are preserved, and those that can't are discarded. After removing the duplicates in the above three datasets, we obtained the substrate and inhibitor datasets with the positive data.

*Negative dataset* Since the substrates collected from the DrugBank and SuperCYP databases are all positive compounds. To obtain the corresponding negative compounds, we borrowed a negative set generation method reported by Yap and Chen in 2005 [25]: firstly, we collected all the drugs in the DrugBank database and deleted the drugs without chemical structures, rare elements and large molecules (molecular weight > 1000 Da). And then, we can randomly select some compounds equal to the number of compounds in the positive set to form a negative set, but do not include the compounds in the positive set. To avoid the uncertainty of random generation, 10 negative sets were randomly generated for each isoenzyme substrate by the above method. As for the enzyme inhibitor, the corresponding negative datasets were collected from the bioassay record “AID 1851” in the PubChem database. The detailed information of modeling data can be seen in Additional files 1 and 2.

#### Validation data

According to the Organization for Economic Co-operation and Development (OECD) principles, not only the internal validation is needed to verify the reliability and predictive ability of models, but also the external validation [26]. Therefore, after internal validation, the chosen models should be further validated by the external dataset to explain their practical predictive ability and generalization ability. Therefore, we proposed the multi-level dataset and external dataset to accomplish this task. The specific data collection process is as follows:

*Multi-level validation set* Based on the definition of DDI in DrugBank: reactions, disturbances or side effects occur when drugs are used in combination, we collected previously reported DDI drug pairs from DrugBank, Physician’s Desk Reference, e-Therapeutics, Medicines Complete and Epocrates RX. All drug pairs were divided into CYP-related drug pairs and possible CYP-related drug pairs according to their annotation. After discarding the drugs without structural information, the remaining drugs were used as the multi-level validation dataset.

*External validation set* In this part, we collected 11 commonly-used drugs that need therapeutic drug monitoring in pharmacy department of Xiangya hospital. And then, we searched their positive CYP450-related metabolic drug interactions in the “Interactions checker” module of the Drugs.com database. After completing their structural information, all drug pairs were prepared as the external validation dataset for further validation. To evaluate the predictive ability of our methodology for negative samples, we thought to find a reliable negative dataset from recent published literatures. Finally, 45,026 reliable negative samples generated by DDI-PULearn were finally collected to further verify the strength of our consensus models. The detailed information of validation data can be seen in Additional file 3.

### Data pretreatment and descriptor calculation

For all the compounds collected in section "Data collection", some pretreatment steps were applied to improve their quality and reliability: all compounds were standardized by the “wash” function of MOE (Molecular Operating Environment software, version 2019, Chemical Computing Group, Montreal, QC, Canada) to disconnect group metals in simple salts, keep only largest molecular fragments, deprotonate strong acids, protonate strong bases and add explicit hydrogens. After that, four types of descriptors were calculated using different cheminformatics tools: 206 two-dimensional descriptors (2D) and 166 MACCS fragments were calculated by the MOE software; 210 CATS descriptors and 1024 ECFP4 fingerprints were calculated by ChemDes and PyBioMed [27]. For these descriptors, two pretreatments were performed to delete some uninformative descriptors before further descriptor selection: (1) delete those descriptors whose variances is 0 or approaches 0; (2) if the correlation coefficient between two descriptors is higher than 0.95, only one was reserved. The chosen descriptors were prepared for further SAR modeling.

### Modeling methods and performance evaluation

In this study, we chose two excellent machine learning approaches to construct the substrate and inhibitor QSAR models for five important CYP450 isozymes: random forest (RF) and the extreme gradient Boosting (XGBoost). RF is an ensemble of unpruned classification or regression trees created by using the bootstrap samples of the training data. Recent studies have showed that RF offers several striking features which make it very attractive for QSAR/QSPR studies including relatively high accuracy of prediction, built-in descriptor selection and a method for assessing the importance of each descriptor to the model [28, 29]. XGBoost belongs to the group of widely used tree learning algorithms and it has two major improvements: (a) speeding up the tree construction and (b) proposing a new distributed algorithm for tree searching. Based on its strengths, XGBoost has become a powerful machine learning tool widely used in data science competitions and industry and provides state-of-the-art results on many problems [30]. For some unbalanced datasets, the constructed models were also unbalanced if the general methods were applied. Therefore, the random sampling method was applied in each modeling process when the numbers of compounds in the positive and negative datasets differ too much and this process was repeated 100 times. After the comparison between models based on different methods and descriptors, a consensus model was finally obtained for further application based on these classification models. The exact hyperparameters of the used ML methods, the model selection method and the data splits method can be found in the supporting information SI6 (Additional file 6). 

To ensure the obtained DDI models have good generalization ability for a new drug pair, Monte-Carlo cross validation was employed to evaluate the model performance. For each dataset, 80% compounds were randomly chosen to build models and the remaining 20% were used as the test set. This process was repeated 100 times and their average values were taken as the assessment indexes. Furthermore, the multi-level datasets and the external dataset were also applied to validate the predictive ability of our models. Five common statistical parameters were used to evaluate the performances of QSAR models: sensitivity (SE), specificity (SP), accuracy (ACC), F value (F), an area under receiver operating characteristic curve (AUC). They are defined as follows:

$$\mathrm{SE}=\frac{\mathrm{TP}}{\mathrm{TP}+\mathrm{FN}}$$

$$\mathrm{SP}=\frac{\mathrm{TN}}{\mathrm{TN}+\mathrm{FP}}$$

$$\mathrm{ACC}=\frac{\mathrm{TP}+\mathrm{TN}}{\mathrm{TP}+\mathrm{FP}+\mathrm{TN}+\mathrm{FN}}$$

$$\mathrm{F}= \frac{2\mathrm{TP}}{2\mathrm{TP}+\mathrm{FP}+\mathrm{FN}}$$

## Results and discussion

### CYP450 isozyme data analysis

As described in the data collection part, we collected the substrate and inhibitor datasets as many as possible for five important CYP450 isozymes, including CYP1A2, CYP2C9, CYP2C19, CYP2D6, and CYP3A4. Their detailed information including the number of active and inactive compounds of substrates and inhibitors are listed in Table 2.

**Table 2:** The detailed information of the modeling datasets

	Substrate		Inhibitor	
	Active	Inactive	Active	Inactive
CYP1A2	198	198	6089	6974
CYP2C9	259	259	4316	8361
CYP2C19	314	314	6027	7135
CYP2D6	357	357	2906	10,826
CYP3A4	792	792	5544	7446

**Fig. 1:**
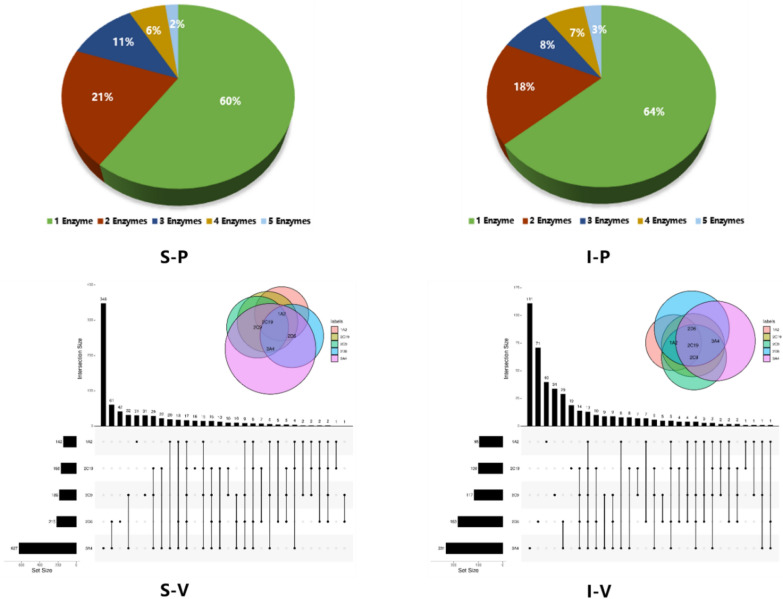
The distribution of the collected drugs and isozymes (S-P, S-V, I-P, and I-V represent the pie diagram of substrates, the UpSet diagram of substrates, the pie diagram of inhibitors, and the UpSet diagram of inhibitors, respectively)

To preliminarily study the complicated relationship between general drugs and five important CYP450 isozymes, we draw the pie charts and UpSet diagrams based on the metabolism information of the drugs collected from the DrugBank and SuperCYP databases. The above pie diagram S-P shows the percentages of the compounds metabolized by different numbers of enzymes and the UpSet diagram S-V represents the number of the compounds metabolized by different enzymes. Specifically, The UpSet diagrams in Fig. 1 were obtained from a web tool, UpSet (https://upset.app/). Different with the most common set visualization approach-Venn Diagrams, UpSet is well suited for the quantitative analysis of data with more than three sets. UpSet plots the intersections of a set as a matrix, as shown in the figure. The top half of this figure is a bar charts for the size of the intersections and a common Venn diagram, which makes the size of intersections easy to compare. In the bottom half, each row corresponds to a set, and the number on the left show the size of the set. Each column corresponds to a possible intersection: the filled-in cells show which set is part of an intersection. From this figure, we can easily see the number of drugs in each intersection and involved isoenzymes and further analysis can be carried out.

From the S-P and S-V diagrams, we could see that 40% of the collected drugs are metabolized by multiple CYP isozymes, and 79% of them (251drugs) are metabolized by 2 or 3 metabolic enzymes. Additionally, we found that CYP3A4 is involved in the metabolic process of 80% of drugs (627 drugs) while CYP1A2 is only involved in the metabolism of 18% of drugs (142 drugs). Similarly, I-P shows the percentages of the compounds that inhibit different numbers of enzymes and I-V represents the number of the compounds that inhibit different enzymes. From these diagrams, we could also find some meaningful things: 36% of drugs can inhibitor multiple CYP isozymes and 73% of them have inhibition effect on 2 or 3 metabolic enzymes. Furthermore, we also found that CYP3A4 can be inhibited by 53% of drugs but CYP1A2 can only be inhibited by 22% of drugs. Based on the above observation, we can draw a conclusion that there is an overlapping relationship between metabolic enzymes and common drugs when considering metabolism and inhibition process, which is also the reason why potential substrates or inhibitors are difficult to be predicted.

### Predictive models based on different machine learning approaches

#### Parameter optimization

As we all know, appropriate parameter has a significant impact on the quality of obtained models and consequently the parameter optimization is a necessary step for model building. Therefore, we have also performed the hyperparameter optimization before modeling process. For the Random Forest (RF) and XGBoost, the grid search method and fivefold cross-validation were applied to optimize a best parameter set for each model. Specifically, for the RF, only one parameter, the number of decision trees (n_tree, from 500 to 2000, interval = 100) was optimized. For the XGBoost, the learning rate (Eta, from 0.01 to 0.3, interval = 0.02), the maximum depth of a tree (maximum depth, from 3 to 10, interval = 1), and the number of models to train in the boosting ensemble (boosting rounds, from 500 to 3000, interval = 500) were optimized. The optimization results were as follows: ntry = 1000; Eta = 0.3; max_depth = 6; boosting rounds = 2000.

#### Predictive models based on random forest

**Fig. 2:**
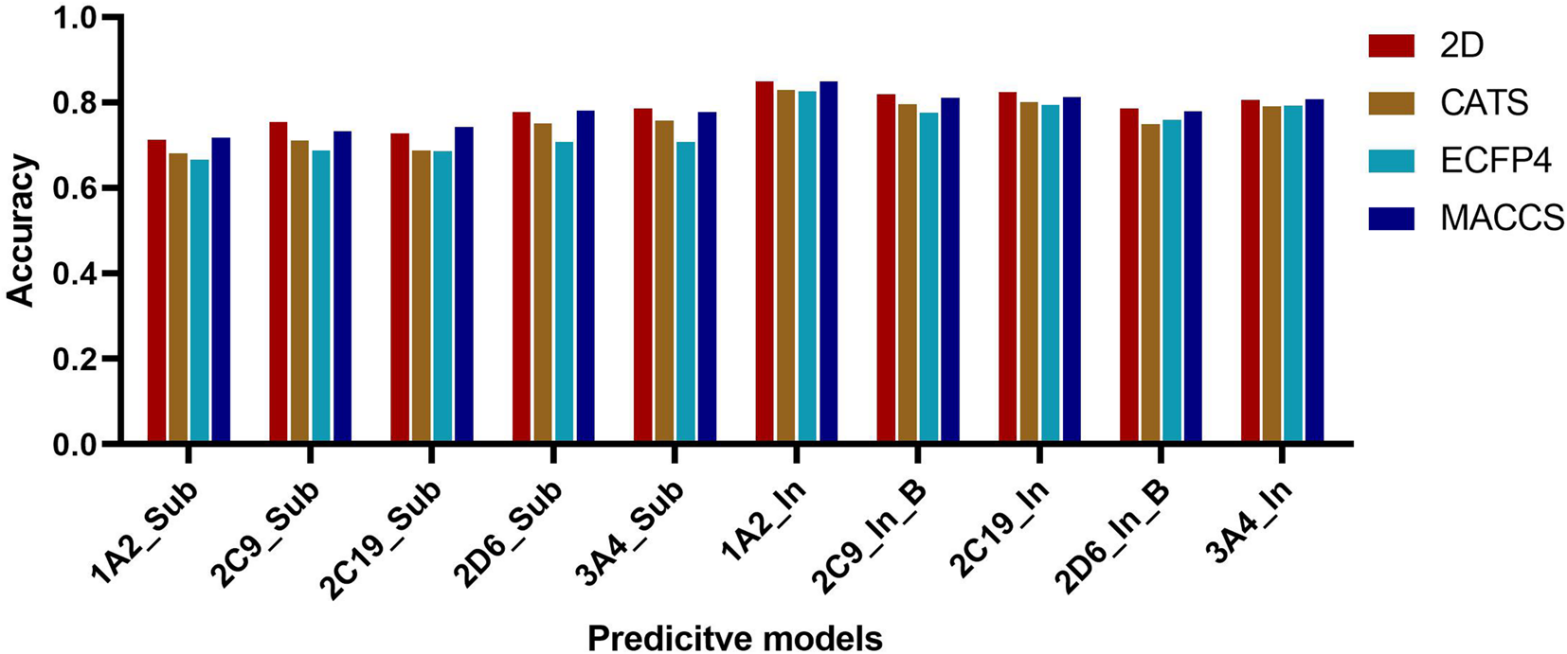
The whole performance of the substrate and inhibitor models using different descriptors

In this part, we constructed the substrate and inhibitor predictive models based on four types of descriptors using RF for five CYP450 enzymes, respectively. For the collected datasets, we totally obtained 5 × 4 × 10 substrate predictive models and 5 × 4 inhibitor predictive models. Each modeling process was repeated for 100 times and their average values were taken as the evaluation parameter of the models. It is noteworthy that for the CYP2C9 and CYP2D6 inhibitor datasets (the number of the negative samples was much larger than that of the positive samples), the random sampling method was applied to obtain the balanced models. The whole statistic performance of the substrate and inhibitor models using different descriptors was shown in Fig. 2. From this figure, we can see that all the substrate models using the 2D descriptor perform better than the others. Based on this, we collected the accuracy values of the substrate models using the 2D descriptor for the 10 randomly-generated negative datasets and listed them in Table 3. Finally, according to their internal validation results, the best models using RF for the substrates and inhibitors were chosen and their detailed predictive ability was listed in Table 4.

**Table 3:** The accuracy values of the substrate models based on the 10 randomly-generated negative datasets

	Des	1A2_Sub	2C9_Sub	2C19_Sub	2D6_Sub	3A4_Sub
1	2D	0.70 ± 0.049	0.72 ± 0.042	0.74 ± 0.045	0.78 ± 0.031	0.75 ± 0.025
2	2D	0.69 ± 0.041	0.74 ± 0.041	0.73 ± 0.050	0.78 ± 0.028	**0.77 ± 0.024**
3	2D	0.71 ± 0.045	0.73 ± 0.041	0.75 ± 0.045	0.78 ± 0.031	0.76 ± 0.023
4	2D	0.69 ± 0.045	0.72 ± 0.048	0.75 ± 0.046	0.78 ± 0.031	0.74 ± 0.020
5	2D	**0.72 ± 0.047**	**0.76 ± 0.035**	0.74 ± 0.047	0.77 ± 0.033	0.76 ± 0.019
6	2D	0.72 ± 0.042	0.71 ± 0.045	0.74 ± 0.049	0.78 ± 0.035	0.75 ± 0.023
7	2D	0.71 ± 0.036	0.74 ± 0.040	0.75 ± 0.045	0.78 ± 0.031	0.75 ± 0.024
8	2D	0.72 ± 0.050	0.73 ± 0.046	0.74 ± 0.051	0.77 ± 0.033	0.75 ± 0.020
9	2D	0.72 ± 0.041	0.73 ± 0.042	**0.77 ± 0.040**	**0.79 ± 0.032**	0.75 ± 0.022
10	2D	0.71 ± 0.046	0.72 ± 0.045	0.73 ± 0.043	0.78 ± 0.034	0.74 ± 0.020

Bold value represents the accuracy of the best of the 10 randomly generated negative sets

**Table 4:** The detailed predictive ability of chosen QSAR models using RF

	Des	SE	SP	F	ACC	AUC
1A2_Sub	2D	0.72 ± 0.067	0.73 ± 0.072	0.72 ± 0.051	0.72 ± 0.047	0.78 ± 0.045
2C9_Sub	2D	0.75 ± 0.060	0.77 ± 0.063	0.75 ± 0.037	0.76 ± 0.035	0.84 ± 0.038
2C19_Sub	2D	0.76 ± 0.063	0.79 ± 0.067	0.77 ± 0.043	0.77 ± 0.040	0.85 ± 0.038
2D6_Sub	2D	0.76 ± 0.046	0.82 ± 0.044	0.79 ± 0.033	0.79 ± 0.032	0.86 ± 0.029
3A4_Sub	2D	0.75 ± 0.038	0.79 ± 0.035	0.76 ± 0.026	0.77 ± 0.024	0.85 ± 0.020
1A2_In	2D	0.82 ± 0.010	0.87 ± 0.009	0.84 ± 0.007	0.85 ± 0.006	0.93 ± 0.005
2C9_In	2D	0.71 ± 0.017	0.90 ± 0.008	0.74 ± 0.011	0.83 ± 0.007	0.90 ± 0.005
2C9_In_B^*^	2D	0.83 ± 0.014	0.81 ± 0.013	0.82 ± 0.010	0.82 ± 0.008	0.89 ± 0.007
2C19_In	2D	0.81 ± 0.010	0.84 ± 0.008	0.81 ± 0.007	0.83 ± 0.006	0.89 ± 0.005
2D6_In	2D	0.46 ± 0.016	0.97 ± 0.017	0.59 ± 0.015	0.87 ± 0.011	0.87 ± 0.009
2D6_In_B^*^	2D	0.74 ± 0.018	0.83 ± 0.018	0.77 ± 0.014	0.79 ± 0.012	0.87 ± 0.011
3A4_In	MACCS	0.73 ± 0.015	0.86 ± 0.009	0.76 ± 0.010	0.81 ± 0.008	0.89 ± 0.006

B^*^ refers to the balanced model using the random sampling method

According to Table 3, we can see that the predictive performance of all the substrate models based on the 10 randomly-generated negative datasets were satisfactory. For the CYP1A2 models, the ACC was in the range of 0.69 ~ 0.72; for CYP2C9, ACC was in the range of 0.71 ~ 0.75; for CYP2C19, the ACC was in the range of 0.73 ~ 0.77; for CYP2D6, the ACC was in the range of 0.76 ~ 0.79, and for CYP3A4, the ACC was in the range of 0.74 ~ 0.76. Therefore, we can draw a conclusion that the accuracies of the substrate models based on the 10 randomly-generated negative sets are very close, indicating that the randomly selected negative sets have little influence on the overall accuracies of the models. According to Fig. 2 and Table 4, we can clearly see that: (1) for the most substrates and inhibitors of CYP450 enzymes (excluding CYP3A4 inhibitor), the QSAR models based on the 2D descriptors performed best for the internal validation; (2) for the substrate models, SE was in the range of 0.71 ~ 0.76, SP was in the range of 0.72 ~ 0.82, the accuracy was in the range of 0.72 ~ 0.79, and AUC was in the range of 0.78 ~ 0.86. Specifically, the whole performances of the CYP2D6 models and CYP3A4 models were better than those of the other two models. Analysis of Fig. 1 and Table 2 illustrates that this may be because the collected data sets of CYP2D6 and CYP3A4 are larger and cover more chemical space than the other data sets; (3) for these inhibitor models, SEs of the final five models are around 0.80, SPs are around 0.85, the accuracy values are around 0.80, and the AUC values are around 0.90. For the CYP2C9 and CYP2D6 datasets, the balanced models performed much better than the unbalanced ones. The 2C9 model is unbalanced: SE is 0.707 and SP is 0.896; 2C9 balanced: SE is 0.828 and SP is 0.810; 2D6 unbalanced: SE is 0.458 and SP is 0.972; 2D6 balanced: SE is 0.736 and SP is 0.834. This change demonstrates that this resampling method we proposed is very effective to correct the model bias and establish a practical balanced model. In whole, these models based on random forest are reliable and robust enough to predict whether a new compound is an inhibitor of the certain CYP450 isoenzymes.

#### Predictive models based on XGBoost and consensus method

As the same as the modeling processes in section "Parameter optimization", we chose another powerful machine learning approach, XGBoost, to construct substrate and inhibitor predictive models. Based on the aforementioned optimal descriptors and negative datasets. The detailed predictive results of the substrate and inhibitor models using XGBoost were listed in Table 5. According to Tables 4 and 5, we can obtain the following information: (1) for the XGBoost models, SE was in the range of 0.72 ~ 0.84, SP was in the range of 0.69 ~ 0.86 and ACC was in the range of 0.70 ~ 0.85; (2) compared with those predictive models using RF, most XGBoost models have similar or even better predictive ability, such as the substrate and inhibitor models for CYP2D6; (3) for all the models based on XGBoost, their SE values were larger than those for the models based on RF no matter their overall accuracy values; and on the contrary, their SP values were always smaller; (4) for the substrate dataset of CYP3A4 isoenzyme, the XGBoost model using the MACCS descriptors performed best but the RF model using the 2D descriptors was the best, which demonstrates the selectivity of the modeling approach to descriptors.

**Table 5:** The detailed predictive ability of the chosen QSAR models using XGBoost

	Des	SE	SP	F	ACC	AUC
1A2_Sub	2D	0.72 ± 0.070	0.69 ± 0.073	0.71 ± 0.046	0.70 ± 0.040	0.77 ± 0.041
2C9_Sub	2D	0.79 ± 0.055	0.73 ± 0.072	0.76 ± 0.042	0.76 ± 0.039	0.83 ± 0.040
2C19_Sub	2D	0.76 ± 0.060	0.74 ± 0.070	0.75 ± 0.048	0.75 ± 0.043	0.82 ± 0.039
2D6_Sub	2D	0.80 ± 0.046	0.79 ± 0.044	0.79 ± 0.034	0.79 ± 0.030	0.86 ± 0.029
3A4_Sub	MACCS	0.77 ± 0.034	0.76 ± 0.037	0.77 ± 0.023	0.77 ± 0.021	0.84 ± 0.018
1A2_In	2D	0.84 ± 0.011	0.87 ± 0.009	0.85 ± 0.007	0.86 ± 0.006	0.93 ± 0.004
2C9_In_B^*^	2D	0.83 ± 0.013	0.80 ± 0.014	0.82 ± 0.009	0.82 ± 0.009	0.89 ± 0.008
2C19_In	2D	0.82 ± 0.011	0.83 ± 0.010	0.81 ± 0.007	0.82 ± 0.006	0.89 ± 0.005
2D6_In_B^*^	2D	0.78 ± 0.018	0.81 ± 0.018	0.79 ± 0.013	0.80 ± 0.012	0.87 ± 0.009
3A4_In	MACCS	0.76 ± 0.011	0.83 ± 0.010	0.77 ± 0.009	0.801 ± 0.007	0.88 ± 0.006

**Fig. 3:**
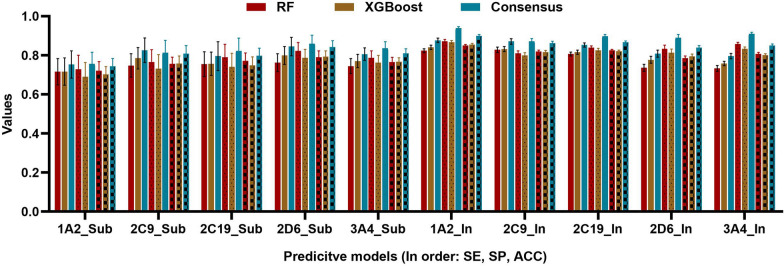
The statistical results of the predictive RF and XGBoost models and the consensus method

Taking into consideration of the different predictive capacity and uncertainty of the models based on RF and XGBoost, the consensus modeling was applied to obtain well-performed predictive models. Consensus modeling can reduce model uncertainty by averaging the outputs from multiple models and can capture the relationship between chemical structures and the endpoint more efficiently than a single model. Thus, a series of consensus models were developed by combining all RF and XGBoost models based on different types of descriptors. The comparison of the statistical parameters (SE, SP and ACC values) for the RF models, the XGBoost models and their consensus models can be seen in Fig. 3. From this figure, we can find the following facts: (1) for all the consensus predictive models, the SE was in the range of 0.70 ~ 0.87, the SP was in the range of 0.75 ~ 0.89 and the ACC was in the range of 0.74 ~ 0.86. (2) On the whole, the predictive power of the consensus models was superior to that of individual models. Especially, for the CYP2C9, CYP2D6 substrate dataset and CYP2D6 inhibitor dataset, the ACC values of their consensus were obviously higher than those of the RF and XGBoost models. (3) For most datasets, the superiority of SE for the consensus model is more obvious than SP. To further prove the usefulness of our consensus method, we performed t-tests and calculated p-values for the results of the consensus models and the individual models. According to the calculated P values, we can see that not only the accuracy but also the SE and SP values between consensus and individual models were different significantly (P < 0.05). That is to say, these consensus models have better prediction capacity to identify potential DDIs based on five important CYP450 isoenzymes and thus play a decisive role in the post marketing pharmacovigilance and drug discovery process. According to the internal validation results of the predictive models based on different methods, we can draw a conclusion that the consensus models perform better than the predictive models based on the RF and XGBoost methods. However, the preliminary conclusion was made only based on the results of the individual inhibitor and substrate models rather than the overall DDI models. Furthermore, considering the OECD principles for model validation, more comprehensive validation is needed to illustrate the practicability and generality of the consensus models.

### Multi-level validation

To further evaluate the predictive ability of the consensus DDI models based on the aforementioned machine learning approaches and large datasets, we additionally collected a multi-level dataset for the external validation. As described in the “data collection” part, we finally collected 1317 positive DDI pairs from several popular databases after deleting duplicates in the training set. To evaluate the reliability of the multi-level validation dataset, we calculated the Tanimoto similarity between the multi-level validation data and the training set compound. ECFP4 fingerprint was applied to represent compounds and the corresponding similarity coefficients were calculated. The results showed that the similarity coefficients between validation set and inhibitor training sets were smaller, generally in the range of 0.05 ~ 0.2. However, the ones between validation set and substrate training sets were larger, mostly in the range of 0.2 ~ 0.6. We suspected that this may be due to that the datasets for CYP inhibitors were much larger than those of CYP substrate. Overall, the multi-level validation dataset was eligible for the evaluation of DDI predictive models. All the drug pairs were divided into three levels according to the following definition: For a DDI pair, drug A is the substrate of a specific enzyme C and drug B is the inhibitor: first level—A and B interact with the same enzyme; second level—A and B interact with the enzyme C; third level—A and B interact with the enzyme C and A is substrate, B is inhibitor. After data processing, the final consensus DDI models were applied to predict the potential DDIs for these drug pairs. The data details and the predictive results were listed in Table 6. According to this table, we can see that the consensus DDI model performed well in three-level validation with the accuracy of 1.000, 0.998 and 0.793 respectively. Except for the CYP3A4 dataset in the third level, the prediction accuracy of the model for other datasets is above 0.8. Therefore, our consensus model also has reliable predictive power when applied for the external compounds.

**Table 6:** The detailed information of the result for the multi-level datasets

	Total	CYP1A2	CYP2C9	CYP2C19	CYP2D6	CYP3A4	Accuracy
First level	1317 (1317)	–	–	–	–	–	1.000
Second level	1310 (1308)	80 (80)	132 (131)	27 (27)	112 (112)	959 (958)	0.998
Third level	1194 (947)	80 (80)	128 (112)	27 (24)	111 (94)	848 (637)	0.793

The numbers outside and inside the parentheses represent the actual and predictive numbers of DDIs respectively

The applicability domain (AD) evaluation is a guarantee for QSAR models in predicting uncertain compounds accurately and reasonably. To estimate the AD of the DDI prediction model, we compared the Tanimoto similarity of drugs that predicted wrong and those predicted right to the training set compounds. For the multi-level validation datasets, there were totally 16 and 14 predicted wrong drugs for the substrate and inhibitor models respectively. In this part, we evaluated the similarity between a drug and the training set by using the average Tanimoto similarity of the 10 compounds most similar to the drug in the training set. After analyzing the similarity results of these drugs, we found that for the substrate predictive models, the Tanimoto similarity values of drugs with wrong predictions were less than 0.15, while the values of drugs with correct predictions were greater than 0.2. And similarly, for the inhibitor predictive models, the average similarity values of these wrong drugs were all below 0.03, while the values of predicted right drugs were larger than 0.05. From a statistical standpoint, these predicted wrong drugs were regarded as outliers of the DDI prediction model and they were distributed outside the application domain of the model. As a result, based on the comparison results of Tanimoto similarity, we can preliminarily assess the application domain of our DDI prediction models. We have reasons to believe that our prediction model may have more reliable prediction results for a new compound whose similarity to the substrate training set compounds is greater than 0.2 and to the inhibitor training set compounds is greater than 0.05.

To find the intrinsic hidden rules in the DDI datasets and further explain the predictive ability of our DDI models, we analyzed the structural features of the compounds in the modeling datasets based on their scaffolds. In this part, the RDKit package was applied to analyze the scaffold of all the compounds. The RDKit package provides a standard decomposition of molecules into scaffolds and carbon skeletons based on the two-dimensional structures of molecules. The scaffold decomposition was proposed by Bemis and Murcko and has become the most widely applied and established scaffold definition. In this definition, scaffolds were extracted from compounds by removing all R-groups but retaining the linkers between ring systems [31]. Based on the scaffolds, Xu and Johnson defined the carbon skeletons in 2002. Carbon skeletons are derived from scaffolds by changing each heteroatom to a carbon atom and all bond orders to single bonds. Thus, different carbon skeletons represent topologically distinct scaffolds [32]. The scaffold and carbon analyses were carried out for the inhibitor and substrate datasets. To provide some valuable information for the readers and the community, not only the Murcko scaffold and carbon scaffold of each dataset but also the similarity/dis-similarity between substrates/inhibitor and non-substrate/non-inhibitors and the most prominent scaffold for each dataset were analyzed in this part. In the compare process of positive and negative drugs, only these scaffolds that appear more than twice were chosen to reduce the occasionality. Table 7 listed the number of scaffolds, the number of carbons and the most prominent scaffolds of each inhibitor and substrate dataset. As shown in Table 7, we can clearly see that no matter the number of skeletons or the number of carbon skeletons, the inhibitor datasets are much larger than the substrate datasets. As we all know, a QSAR model derived from structurally diverse compounds will generally cover a large chemical space and consequently have a wide application domain. And that’s why the predictive ability of the inhibitor models was better than that of the substrate models. Even so, all the five substrate datasets still have relatively diverse chemical skeletons which covered almost all the chemical structures commonly appeared in drug compounds. Based on the further comparation of scaffolds that appear more than twice, some interesting clues hidden in the each CYP isoenzyme dataset were found: (1) CYP1A2: For CYP 1A2 inhibitor, there were 96 identical scaffolds in the positive and negative datasets and the top three (frequency) were 4, 1 and 168 (Murcko class). According to the explanation document of Murcko class, three scaffolds were benzene ring, pyridine ring and benzylaniline respectively and they are common elements for the inhibitor of CYP1A2. Addition to it, the positive and negative datasets contain 658 and 771 unique scaffolds, respectively. Among them, class 1526, 3567, 3606 were the most prominent ones for CYP1A2 inhibitor, while class 54, 7220, 5, 414, 849 were important for non-inhibitors. Based on the explanation document, that is to say, drugs or chemicals with 2-Phenylquinazoline, *N*-Benzyl-5-phenyl-pyrimidin-4-amine, *N*-benzyl-2-phenylpyrimidin-4-amine and without Piperidine ring, Imidazole ring, 9-(Tetrahydrofuran-2-yl)-9H-purine, or 3-Benzyl-3,9-diazaspiro [5, 5] undecane were probably CYP inhibitors. Similarly, for the substrate, cyclopropane and benzyl-[2-(2-phenoxyethoxy) ethyl] azanium were the same scaffolds in the positive and negative datasets and compounds with 4-*N*-thiazole, cyclohexane were more likely to be CYP1A2 substrates. (2) CYP2C9: For the inhibitor, the benzene ring, triazine ring and 6-Benzylaminopurine were the universal substructures in the positive and negative datasets and drugs with quinoline furan, and benzothiazole were more probably to be inhibitors. And for the substrate, the diphenylborane was the most common scaffold in the positive and negative datasets and the compounds with 1-(14-quinolin-1-ium-1-yltetradecyl) quinolin-1-ium, 4-*N*-thiazole or [1, 3] benzodioxolo [5,6-c] phenanthridin-12-ium were supposed to be the substrates. (3) CYP2C19: For its inhibitor, the benzene ring, indole ring and diphenylmethane were common scaffolds in the positive and negative datasets and 3,9-diazaspiro [5.5] undecane and cyclopropylphenylmethane may be used for the identification of inhibitor and non-inhibitor. For the substrate, phenazoline and 1-(1,3-Benzodioxol-5-Ylmethyl) Piperidine were the most frequent identical scaffolds for the positive and negative datasets and compounds with 1-Phenylpiperazinium, Tryptoline and Phenylbutylpyrrolidine were more like to become substrates. (4) CYP2D6: For the inhibitor, the benzene ring, 4-*N*-thiazole and benzimidazole were the same scaffolds in the positive and negative datasets and the scaffolds pyridazine, 2-phenyl-*N*-(pyridin-3-ylmethyl) pyrimidin-4-amine, (1-methylpyrrol-2-yl)-[2-(4-phenylphenyl)-2,9-diazaspiro [5.5] undecan-9-yl] methanone were the most potential scaffolds to distinguish the inhibitors. For the substrate, the pyridine ring and 4,4-diphenylimidazolidine were the same scaffolds in the positive and negative datasets and the compounds with 4-anilinoquinazoline, 1,3,5-triazine, 9-anilinoacridine were the most probably ones to be substrates. (5) CYP3A4: For the inhibitor, there are 108 same scaffolds both in the positive and negative datasets and the most frequent three in order were the benzene ring, the pteridine ring and the *N*-benzylaniline. The most prominent scaffolds for inhibitors were 6-(1,3-thiazol-4-yl)-3,4-dihydro-2H-1,4-benzoxazine and 2-(furan-3-yl)-*N*-phenylquinazolin-4-amine. For the substrate, 1,6-Dihydropurine, 3,4-Diindolyl pyrrole were the most important identical scaffolds for the positive and negative datasets and the scaffolds Tryptoline and 1-tritylimidazole were probably the most informative skeletons to identify substrates and non-substrates for CYP3A4. Moreover, we can also find some indetectable facts for the prediction of substrate and inhibitors: (1) the benzene ring was the most common scaffold for all the inhibitor datasets, no matter it was positive or negative. And the second one is *N*-benzylaniline; (2) the scaffold of *N*-benzyl-5-phenylpyrimidin-4-amine was the important skeleton for the non-inhibitor of CYP2C19 and the inhibitor of CYP1A2; (3) compounds with the scaffold of 4-*N*-thiazole could be a substrate of CYP1A2 and CYP2C9, and compounds with the scaffold of Tryptoline could be a substrate of CYP2C19 and CYP3A4. Based on the above statements and discussion, we hope to provide some basis and reference for further research in the future and we believe that the consensus DDI models constructed in this study are robust and reliable enough for further application in drug discovery and clinical practices. The explanation document of Murcko class mentioned in the article can be found in the Additional file 5.

**Table 7:** The scaffold analysis results of the substrate and inhibitor datasets

Subsets			Number of scaffolds	Number of carbons	Most prominent scaffolds (Murcko Class)	Common scaffolds
1A2	Inhibitor	Positive	3520	1418	1526, 3567, 3606	4, 1, 168
		Negative	4257	2090	54, 7220, 5, 414, 849	
	Substrate	Positive	150	94	36, 8, 14	3, 56
		Negative	163	113	158, 43	
2C9	Inhibitor	Positive	3088	1708	4866, 4863, 4885	4, 3366, 179
		Negative	4353	1793	59, 3303, 3413	
	Substrate	Positive	168	119	66, 36, 70	19
		Negative	166	123	173, 199, 254	
2C19	Inhibitor	Positive	4189	2015	3756, 188, 349, 5176	4, 6, 39
		Negative	3751	1614	3567, 461, 3570, 7237	
	Substrate	Positive	132	89	7, 31, 63	47, 16
		Negative	137	105	93	
2D6	Inhibitor	Positive	1836	1003	74, 3742, 3904, 3911	4, 36, 1655
		Negative	6244	2580	3718, 3744, 56	
	Substrate	Positive	209	150	40, 42, 45	1, 26
		Negative	223	153	212, 62, 38	
3A4	Inhibitor	Positive	3536	1912	3504, 3539, 3592	4, 3576, 168
		Negative	4227	1630	58, 429, 7136	
	Substrate	Positive	518	385	57, 31, 80	38, 8, 60
		Negative	506	301	541, 530, 533, 105, 631	

### External validation

After multi-level validation, we collected an external dataset to further evaluate the predictive ability of the models as described in the “data collection” part. In this part, 11 commonly-used drugs that need therapeutic drug monitoring in Xiangya hospital were chosen for the external validation (Carbamazepine, Oxcarbazepine, Phenytoin sodium, Phenobarbital, Valproic acid, Methotrexate, Voriconazole, Vancomycin, Tigecycline, Meropenem, and Imipenem). Due to their nonlinear pharmacokinetic properties or low therapeutic index, patients need to regularly monitor if their concentrations and potential DDIs lead to serious consequences. Therefore, it is of great value to predict possible DDIs for them. Totally, we collected 132 knowns positive DDIs from the Drugs.com database after searching the aforementioned drugs and deleting duplicates in the training set, and the final consensus DDI models were applied to evaluate them. To study the reliability of the external datasets, Tanimoto similarity was proposed to evaluate the similarity between external and training sets. In this part, ECFP4 fingerprint was applied to represent the chemical compounds and then Tanimoto similarity values were calculated between them. The results showed that the Tanimoto similarity coefficients between external test sets and inhibitor training sets were smaller, generally below 0.2. However, the ones between validation set and substrate training sets were larger, most are around 0.5. Overall, the external test dataset was reliable enough to evaluate our obtained consensus models. According to the prediction results, the number of the true positives was 105, the number of false negatives was 27, and the accuracy of the DDI model reached 79.50%. Moreover, to evaluate the predictive ability of our methodology for negative samples, a new dataset composed of reliable negative DDIs was necessary in our validation procedure. As we all know, DDI prediction is now facing challenges due to the lack of experimentally verified negative samples, and thus we thought to find a reliable negative dataset from recent published literatures. In this part, 45,026 reliable negative samples generated by DDI-PULearn were finally collected to further verify the strength of our consensus models [33]. According to the result, 81.94% of negative samples were classified correctly by the final model and this newly added validation demonstrated that our proposed models were effective enough for both positive and negative DDI samples. On the whole, these prediction results of the external validation proved the usefulness and reliability of our final model and consequently provided a theoretical basis for its practical application in the DDI prediction of unknown drugs.

### Compared with other advanced methods

To further test the reliability and robustness of our DDI prediction model, we decided to compare it with other advanced methods. Considering the pharmacokinetics mechanism of our study and the dataset availability of published literatures, two recently methods were chosen to compare with ours: The first one is a Multitask Deep Autoencoder Neural Network method (DNN) to predict Human Cytochrome P450 Inhibition, proposed by Pei in 2018 [34]. In this paper, based on a dataset containing 13,000 compounds, they developed a multitask model for concurrent inhibition prediction of five major CYP450 isoforms, namely, 1A2, 2C9, 2C19, 2D6, and 3A4. The other one is the stratified bagging (SB) method used in Xin Xu’s study recently [35], which was applied to develop quantitative structure–activity relationship models for the prediction of CYP2C9, CYP2D6, and CYP3A4 Catalysis and Inhibition. To validate the effectiveness of as many models as possible, two external datasets were collected for further evaluation respectively: the multi-validation dataset proposed in Part 4.3 of this manuscript was used to compare our models with Pei’s DNN models; the National Center for Advancing Translational Sciences (NCATS) dataset was collected from Xu’s publication and used to compare our models with SB models. The detailed comparison results (Accuracy) between models using different methods were listed in the below Table 8. From this table, we can see that for the inhibitors of multi-validation dataset, the accuracy values of DNN models were in the range of 0.3 ~ 0.7 while our models resulted accuracy values in the range of 0.8 ~ 1.0. Although the DNN model reported an accuracy of 0.8 for external test dataset, its predictive power does not appear to be very good for this validation dataset. Given that all compounds in this dataset are positive, we can only draw a preliminary conclusion that our model is better than this DNN model at predicting positive data. As for the NCATS dataset, it contains not only positive but also negative samples for CYP inhibitor and substrate. After predicting the NCATS dataset, the accuracy values of SB models and ours were both in the range of 0.5 ~ 0.7. In detail, except for the dataset of 3A4_Sub, our models performed equal or better for all the other datasets than the SB models. Based on the comparison results between two recently published models and ours, we have reasons to believe that the prediction models derived from this study were reliable and effective enough for the potential metabolic DDI screening in the future.

**Table 8:** The detailed comparison results (Accuracy) between models using different methods

Dataset	Methods	1A2_In	2C9_In	2C19_In	2D6_In	3A4_In	2C9_Sub	2D6_Sub	3A4_Sub
Multi-validation	DNN	0.293	0.345	0.471	0.712	0.506	–	–	–
	Ours	1.000	1.000	1.000	0.856	0.915	–	–	–
NCATS	SB	–	0.614	–	0.550	0.653	0.618	0.607	0.663
	Ours	–	0.662	–	0.629	0.664	0.632	0.608	0.547

**Table 9:** The most frequently 10 drugs predicted to cause DDIs when interacting with other drugs

Drugs	Interact with	No. of predicted DDIs
Cidofovir	CYP2C9, CYP3A4	455
Alendronate	CYP1A2, CYP3A4	444
Trifluridine	CYP2D6, CYP3A4	442
Promazine	CYP2C19, CYP2D6, CYP3A4	439
Vinblastine	CYP3A4	424
Chloropyramine	CYP2C19, CYP2D6	397
Citalopram	CYP2C19, CYP2D6, CYP3A4	396
Clarithromycin	CYP3A4	384
Vincristine	CYP3A4	384
Nitrendipine	CYP3A4	384

### Application of DDI models

After evaluation, our DDI models were proved to be reliable and practical. To broaden the application of these models, we decided to apply our models to predict the potential DDIs for currently marketed drugs. For this propose, we searched for the FDA-approved mono-component electroneutral organic drugs in the DrugBank and thus 1132 drugs were extracted. In total, there are 1132 × 1132/2 = 640,146 possible binary combinations for these drugs. After predicting all possible combinations, we proposed a new index, P_DDI_, to screen the potential DDI drug pairs and it was defined as follows: P_DDI_ = P_drug 1_ × P_drug 2_, where P_drug 1_ and P_drug 2_ are the predictive probabilities of drug 1 and drug 2, respectively. As a result, we found 54,013 potential drug pairs according to their predictive possibilities (the P_DDI_ threshold was defined at 0.95). For each CYP isozyme, the specific potential DDI number is as follows: 3328 drug pairs for CYP 1A2, 4415 drug pairs for CYP 2C9, 5935 drug pairs for CYP 2C19, 10,749 drug pairs for CYP 2D6 and 38,805 drug pairs for CYP 3A4. According to these results, we can see that some drug pairs have potential DDIs based on multi-enzymes and DDIs are more likely to occur for drugs that interact with CYP3A4. After checking the number of the DDIs for each drug, the most frequently 10 drugs predicted to cause unsafe DDIs when interacting with other drugs are listed in Table 9. From this table, we can see that most of the 10 drugs interact with more than one enzyme and nine of them are associated with CYP3A4. Oppositely, only Cidofovir may have the interaction with CYP2C9. Detailedly, Cidofovir and Trifluridine are injectable antiviral medication for the treatment of cytomegalovirus (CMV) retinitis in patients with acquired immune deficiency syndrome (AIDS) and primary keratoconjunctivitis, respectively [36, 37]. Vinblastine and Vincristine are antitumor vinca alkaloid isolated from Vinca Rosea and the CYP3A subfamily facilitates their metabolism [38]. Among the remaining 4 drugs, Chloropyramine and Citalopram belong to the first-generation antihistamine drug and antidepressant agent, respectively, and both of them have interaction with CYP2C19 and CYP2D6 [39, 40]. Moreover, Clarithromycin and Nitrendipine mainly interact with CYP3A4 [41, 42]. Based on the above analyses, clinician and clinical pharmacist should avoid the combination of the above drugs when prescribing to avoid serious adverse reactions caused by drug interactions. All the related information of can be found in Additional file 4 (Table 9).

## Conclusion

In this study, we took mechanism-specific metabolic DDIs caused by Cytochrome P450 as the breakthrough point, RF and XGBoost were used to construct the computational models based on 4 different descriptors (2D, CATS, ECFP4, and MACCS) for substrates and inhibitors of five important CYP450 isoenzymes. The predictive ability differences between the inhibitor and substrate models using RF and XGBoost demonstrate that the models based on the datasets with more chemical skeletons and optimal modeling methods have a more wider application domain and thus the derived DDI models were more reliable and practical in the future applications. To reduce the model uncertainty, a series of consensus models were constructed by combining RF and XGBoost models. For the internal validation, the whole accuracy and AUC value of the final DDI model was around 0.8 and 0.9, respectively. When it was applied to the external datasets, its accuracy was 0.793 and 0.795 for the multi-level validation and external validation, respectively. Additionally, a series of evaluation including AD assessment, the scaffold analysis and comparison with recent published models were carried out to prove the reliability and effectiveness of our models. Finally, we applied our model to predict the FDA-approved drugs and found some drug pairs with potential DDIs. In conclusion, we constructed a practical and reliable DDI predictive model based on the CYP450 enzyme family and aimed to help clinicians avoid high-risk drug combinations in prescribing, help drug researchers assess potential DDI quickly and accurately in the early stages of development and provide valuable references for the subsequent studies and findings of CYP450-related drug–drug interactions.

## Supplementary Information

**Additional file 1.** The final positive DDIs data and selected negative DDIs data with preprocessed descriptors.

**Additional file 2.** The randomly generated negative datasets in the modeling process.

**Additional file 3.** Additional datasets for multi-level validation and external validation.

**Additional file 4.** The FDA-approved drugs for validation.

**Additional file 5.** The explanation document of Murcko class for the scaffold analysis.

**Additional file 6.** The exact hyperparameters of the used ML methods, the model selection method and the data splits method.

## Data Availability

All the datasets supporting the conclusion of this article are available in Additional files.

## Abbreviations

DDI: Drug–drug interaction
CYP450: Cytochrome P450
QSAR: Quantitative structure–activity relationships
2D: Two-dimensional
RF: Random forest
XGBoost: Extreme gradient Boosting
SE: Sensitivity
SP: Specificity
ACC: Accuracy
F: F value
AUC: Area under receiver operating characteristic curve
