# Supplementary material for: Machine learning to predict metabolic drug interactions related to cytochrome P450 isozymes
Source: J Cheminform. 2022 Apr 15;14:23. doi: 10.1186/s13321-022-00602-x (PMC9013037; doi:10.1186/s13321-022-00602-x)
Supplement: Supplementary file 5 — Additional file 5. The explanation document of Murcko class for the scaffold analysis. [file 13321_2022_602_MOESM5_ESM.docx]

| **The explanation document of Murcko class for scaffold analysis** | | |
| --- | --- | --- |
| **Murcko class** | **Framework** | **Chemical structure** |
| 1 | n1ccccc1 | 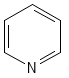 |
| 3 | C1CC1 | 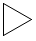 |
| 4 | c1ccccc1 | 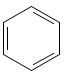 |
| 5 | [nH]1cncc1 | 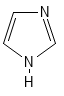 |
| 6 | [nH]1c2c(cc1)cccc2 | 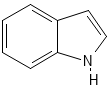 |
| 7 | N1(c2ccccc2)CC[N+H2]CC1 | 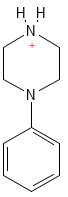 |
| 8 | c1(-c2ccccc2)[nH]c2c(n1)NCNC2 | 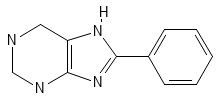 |
| 14 | C1CCCCC1 | 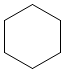 |
| 16 | C(N1CCCCC1)c1cc2OCOc2cc1 | 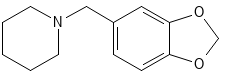 |
| 19 | B(c1ccccc1)c1ccccc1 | 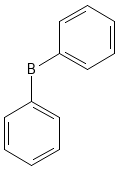 |
| 26 | c1(C2(c3ccccc3)NCNC2)ccccc1 | 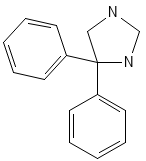 |
| 31 | [nH]1c2c(c3c1CNCC3)cccc2 | 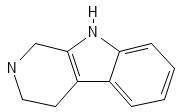 |
| 36 | s1cnnc1 | 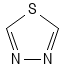 |
| 38 | [nH]1c2NC=NCc2nc1 | 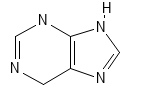 |
| 39 | C(c1ccccc1)c1ccccc1 | 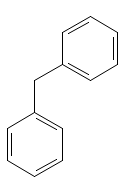 |
| 40 | N(c1ncnc2c1cccc2)c1ccccc1 | 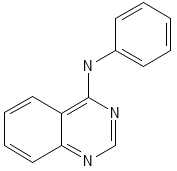 |
| 42 | n1cncnc1 | 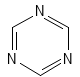 |
| 43 | C12CC3CC(C1)CC(C2)C3 | 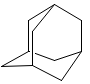 |
| 45 | N(c1c2c(nc3c1cccc3)cccc2)c1ccccc1 | 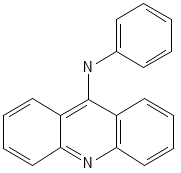 |
| 47 | N(Cc1ccccc1)(CC1=NCCN1)c1ccccc1 | 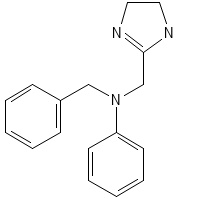 |
| 54 | N1CCCCC1 | 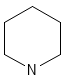 |
| 56 | O(CCOc1ccccc1)CC[N+H2]Cc1ccccc1 | 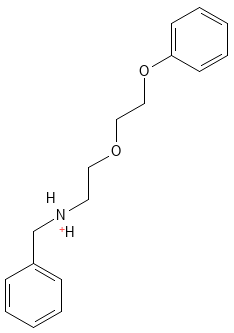 |
| 57 | S(Cc1ccccc1)CC1=N[S+2H2]c2c(N1)cccc2 | 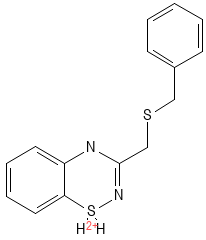 |
| 58 | O1c2c(OC1)cc1c(-c3[n+](cc4c(c3)cccc4)CC1)c2 | 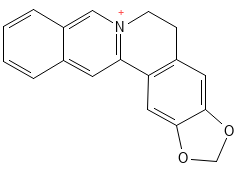 |
| 59 | C(c1ncccc1)(c1ccccc1)c1ccccc1 | 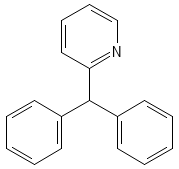 |
| 60 | c1(C2=C(c3c4c([nH]c3)cccc4)CNC2)c2c([nH]c1)cccc2 | 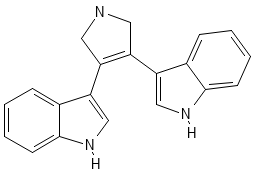 |
| 62 | c1(C2=NCCN2)nc2c(cc1)cccc2 | 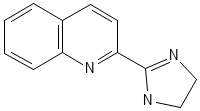 |
| 63 | C(CCc1ccccc1)CN1CCCC1 | 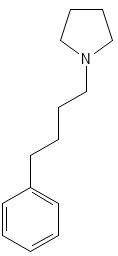 |
| 66 | C(CCCCCCC[n+]1c2c(ccc1)cccc2)CCCCCC[n+]1c2c(ccc1)cccc2 | 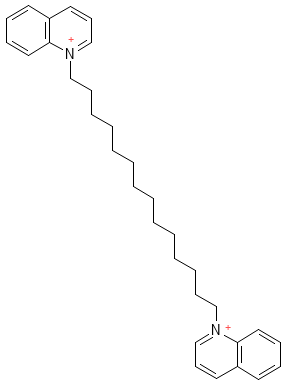 |
| 70 | O1c2c(OC1)cc1c(c3[n+H]cc4c(c3cc1)cccc4)c2 | 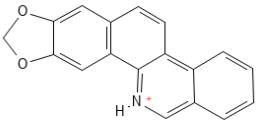 |
| 74 | O1c2c(OC1)cc1c(NN=CC1)c2 | 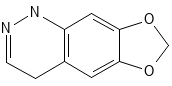 |
| 80 | C(n1cncc1)(c1ccccc1)(c1ccccc1)c1ccccc1 | 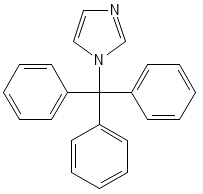 |
| 93 | [i+]1c2c(c3c1cccc3)cccc2 | 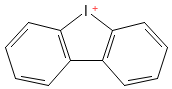 |
| 105 | n1(-c2ncccn2)nccc1 | 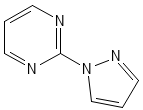 |
| 158 | [S+2H2]1c2c(cccc2)CCC1 | 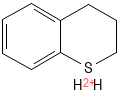 |
| 168 | N(Cc1ccccc1)c1ccccc1 | 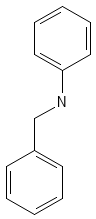 |
| 173 | O(N=C(c1ccccc1)c1ccccc1)CCN1CC=CCC1 | 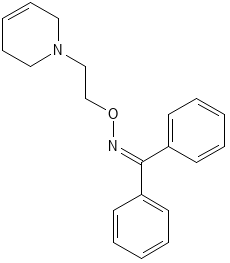 |
| 179 | N(Cc1ccccc1)c1ncnc2[nH]cnc12 | 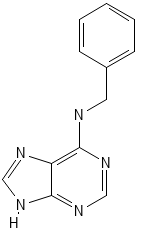 |
| 188 | [S+2H2](Nc1sccn1)c1ccc(NCc2ccccc2)cc1 | 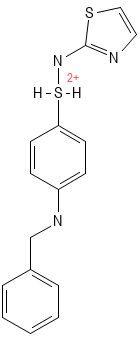 |
| 199 | C(N1CCN(c2nc3c(cn2)cccc3)CC1)c1occc1 | 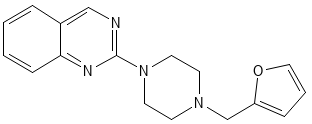 |
| 212 | c1(-c2ccccc2)scnc1 | 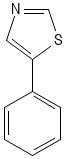 |
| 254 | N(c1ncncc1)c1ccccc1 | 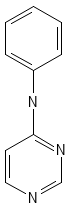 |
| 349 | O(CC12CC3CC(C1)CC(C2)C3)CC1OC(N2C=CC=NC2)CC1 | 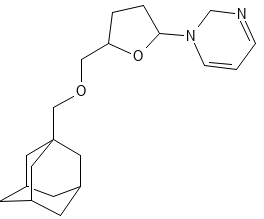 |
| 414 | n1(C2OCCC2)c2ncncc2nc1 | 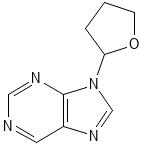 |
| 429 | o1c2c(c3c1CCCC3)cccc2 | 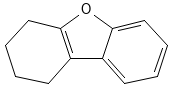 |
| 461 | [nH]1cccc1 | 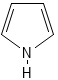 |
| 530 | C(Cc1[nH]c2c(n1)NCNC2)Cc1[nH]c2c(n1)NCNC2 | 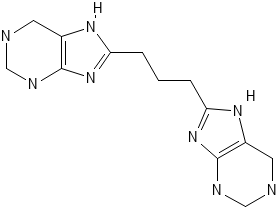 |
| 533 | C(n1c2c(c3c1cccc3)cccc2)n1c2c(c3c1cccc3)cccc2 | 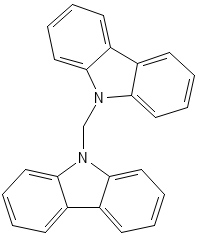 |
| 541 | S(CSc1sc2c(n1)cccc2)c1sc2c(n1)cccc2 | 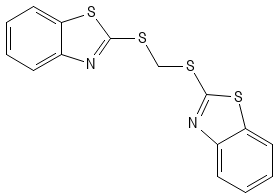 |
| 631 | N(=Cc1nc2c(cc1)cccc2)c1cnc2c(c1)cccc2 | 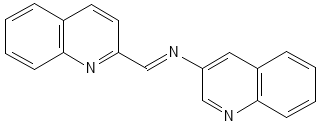 |
| 849 | C(N1CCC2(CC1)CCNCC2)c1ccccc1 | 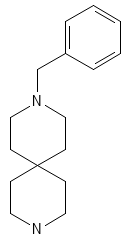 |
| 1526 | c1(-c2ccccc2)nc2c(cn1)cccc2 | 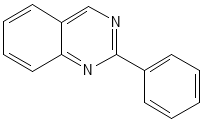 |
| 1655 | N(Cc1ccccc1)C1=CNN(c2ccccc2)C1 | 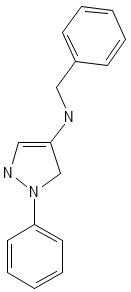 |
| 3303 | S(C=NCCc1ccccc1)CCCc1[nH]cnc1 | 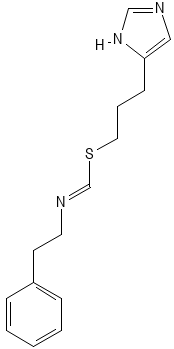 |
| 3366 | N(CCC1=CCCCC1)c1nc(N2CCOCC2)ncn1 | 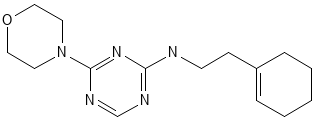 |
| 3413 | S(CCNc1sccn1)c1n(CCNc2ccccc2)nc(-c2ccncc2)n1 | 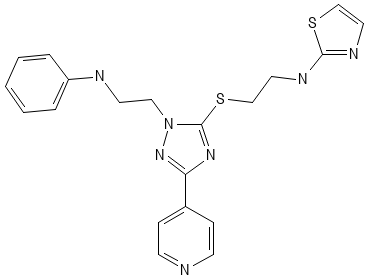 |
| 3504 | N(Cc1nnsc1)(Cc1ccncc1)c1ccccc1 | 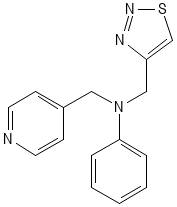 |
| 3539 | c1(-c2cc3NCCOc3cc2)ncsc1 | 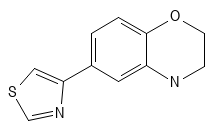 |
| 3567 | N(Cc1ccccc1)c1c(-c2ccccc2)cncn1 | 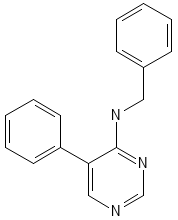 |
| 3570 | c1(-c2conc2)ncncc1 | 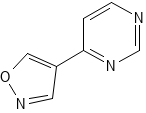 |
| 3576 | N(c1nc2N(C3CC3)CC=Nc2cn1)c1ccccc1 | 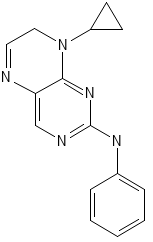 |
| 3592 | N(c1nc(-c2cocc2)nc2c1cccc2)c1ccccc1 | 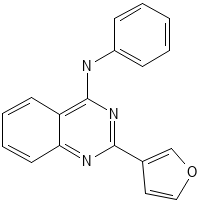 |
| 3606 | N(Cc1ccccc1)c1nc(-c2ccccc2)ncc1 | 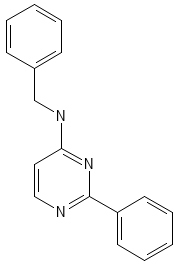 |
| 3718 | C(N1CC2(CN(Cc3nccnc3)CCC2)CC1)c1sccn1 | 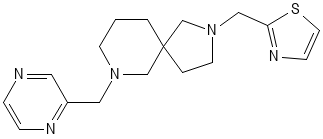 |
| 3742 | N(Cc1cnccc1)c1nc(-c2ccccc2)ncc1 | 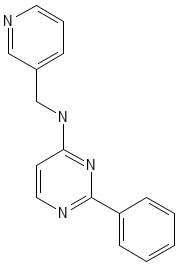 |
| 3744 | C(N1CCC2(CC1)CCN(c1ccccc1)CC2)c1ccncc1 | 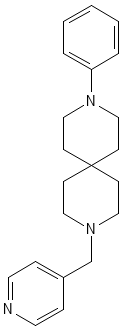 |
| 3756 | C12(CCNCC1)CCNCC2 | 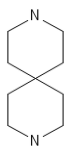 |
| 3904 | C(N1CC2(CN(Cc3ccccc3)CC2)CCC1)c1nnsc1 | 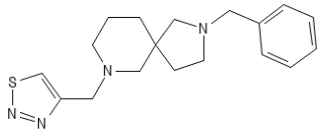 |
| 3911 | C(N1CCC2(CN(c3ccc(-c4ccccc4)cc3)CCC2)CC1)c1[nH]ccc1 | 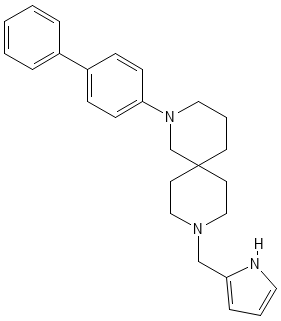 |
| 4863 | [S+2H2](NC(CNc1ncccc1)c1ccccc1)c1c2nsnc2ccc1 | 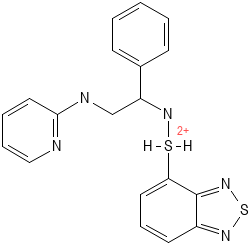 |
| 4866 | C(N1CCCCC1)c1oc2nc3c(cc2c1)cccc3 | 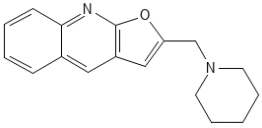 |
| 4885 | C12(OC3C(NCCC3)C1)C=C1C(C3C(C4C(=CC3)CCCC4)C1)CC2 | 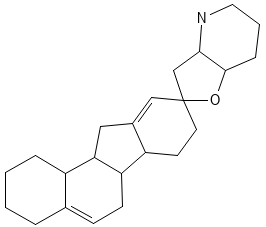 |
| 5176 | C(c1ccccc1)C1CC1 | 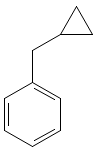 |
| 7136 | O(CN1CCCCC1)CC1C(Oc2ccccc2)C=CC(c2ccccc2)O1 | 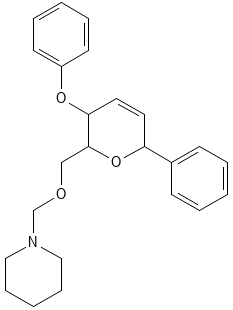 |
| 7220 | N1CC2C3C(CCC2C1)CCCC3 | 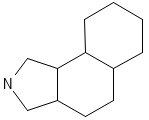 |
| 7237 | O(N=C1C2OC2CC2N3N(CN(c4ccccc4)C3)CCC12)CCN1c2c(nccc2)CCCC1 | 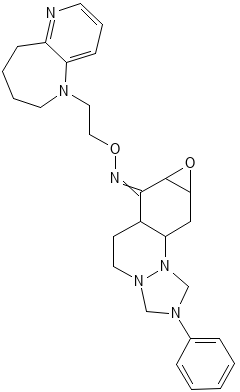 |
