# Supplementary material for: Machine learning to predict metabolic drug interactions related to cytochrome P450 isozymes
Source: J Cheminform. 2022 Apr 15;14:23. doi: 10.1186/s13321-022-00602-x (PMC9013037; doi:10.1186/s13321-022-00602-x)
Supplement: Supplementary file 6 — Additional file 6. The exact hyperparameters of the used ML methods, the model selection method and the data splits method. [file 13321_2022_602_MOESM6_ESM.docx]

**1. The exact hyperparameters of the used ML methods**

For the Random Forest (RF) and XGBoost, the grid search method and 5-fold cross-validation were applied to optimize a best parameter set for each model. Specifically, for the RF, only one parameter, the number of decision trees (n_tree, from 500 to 2000, interval =100) was optimized. For the XGBoost, the learning rate (Eta, from 0.01 to 0.3, interval = 0.02), the maximum depth of a tree (maximum depth, from 3 to 10, interval=1), and the number of models to train in the boosting ensemble (boosting rounds, from 500 to 3000, interval=500) were optimized. The optimization results were as follows:

RF: ntry=1000;

XGBoost: Boosting rounds=2000;

Eta=0.3;

max_depth=6.

**2. The data splits and model selection method**

In this manuscript, we chose the best models based on their predictive power. To evaluate the predictive performance of obtained models, the Monte-Carlo cross validation was employed to evaluate the model performance. For each evaluation process, the data splits method was as follows: 80% compounds were randomly chosen to build models and the remaining 20% were used as the test set. This process was repeated 100 times and their average accuracy values were taken as the assessment indexes to select the best ones for final application.

Five common statistical parameters were used to evaluate the performances of QSAR models: sensitivity (SE), specificity (SP), accuracy (ACC), F value (F), an area under receiver operating characteristic curve (AUC).
